# Improving the Mechanical Properties of Fly Ash-Based Geopolymer Composites with PVA Fiber and Powder

**DOI:** 10.3390/ma15072363

**Published:** 2022-03-23

**Authors:** Jianchen Cai, Jinyun Jiang, Xiang Gao, Meiya Ding

**Affiliations:** College of Mechanical Engineering, Quzhou University, Quzhou 324000, China; jiangjinyun2@163.com (J.J.); A17535873277@163.com (X.G.); ding1600615038@163.com (M.D.)

**Keywords:** geopolymer composites, polyvinyl alcohol, mechanical strength, toughening mechanism

## Abstract

In this work, polyvinyl alcohol (PVA) fiber and powder were added to geopolymer composites to toughen fly ash-based geopolymer, and their different toughening mechanisms were revealed. Firstly, different contents of active granulated blast furnace slag (GBFS) were added to the geopolymer to improve the reactivity of the GBFS/fly ash-based geopolymer, and the best ratio of GBFS and fly ash was determined through experiments testing the mechanical properties. Different contents of PVA powders and fibers were utilized to toughen the geopolymer composites. The effect of the addition forms and contents of PVA on the mechanical properties, freeze–thaw cycle resistance, and thermal decomposition properties of geopolymer composites were systematically studied. The results showed that the toughening effect of PVA fiber was better than that of PVA powder. The best compressive strength and flexural strength of geopolymer composites toughened by PVA fiber were 41.11 MPa and 8.43 MPa, respectively. In addition, the composition of geopolymer composites was explored through microstructure analysis, and the toughening mechanisms of different forms of PVA were explained. This study provided a new strategy for the toughening of geopolymer composites, which can promote the low-cost and efficient application of geopolymer composites in the field of building materials.

## 1. Introduction

The geopolymer was firstly proposed by French scientist Davidovits [1]. It was an environmentally friendly material, made of fly ash, slag, and industrial solid waste [2,3]. Compared with ordinary Portland cement (OPC), it can not only reduce greenhouse gas emissions, but also recycle a large amount of industrial waste. Previous studies [4,5] have shown that, compared with production of OPC, the fly ash-based geopolymer production needs approximately 60% less energy, and CO_2_ emissions are reduced by at least 80%. It was also considered that the geopolymer has the advantages of high strength, good freeze–thaw cycle, and great acid and alkali corrosion resistance [6,7]. Extensive research on the geopolymer has been carried out worldwide, hoping to promote geopolymer composites as the ultimate sustainable building material in the future [8,9]. Although geopolymer has great development potential in the construction industry, its low brittle behavior is one of the limitations of its application [10,11]. Many researchers have carried out studies on how to improve the toughness of geopolymer.

One of the methods is to add fiber to geopolymer, which can improve the mechanical properties of materials, especially the flexural strength, and reduce the propagation of microcracks in materials [12,13,14]. Yan et al. [15] provided an effective and simple method to prepare the carbon fiber felt-reinforced geopolymer composites and improve the flexural strength. Zhang et al. [16] showed that the addition of chopped carbon fibers in the geopolymer provided effective crack control mechanism under high-temperature exposure. Shaikh [17] prepared alkali-activated low-calcium fly ash-based geopolymer by adding 2 wt.% steel fiber, the results showed that the bending hardening and multiple crack behavior of the fiber-reinforced geopolymer composites were similar to that of cement-based system. High-strength reinforcing materials such as carbon fiber and steel fiber can indeed significantly improve the toughness of polymers, but the high cost limited their large-scale application

Polymer fiber was one of the most promising reinforcement materials for large-scale applications [18]. Shaikh [19] compared the compressive strength PP fiber-reinforced geopolymer and PET fiber-reinforced geopolymer under two different curing methods, and the results showed that the compressive strength of the two fiber-reinforced geopolymer was better than that of cement composites. Zhang [20] prepared short PVA fiber-reinforced fly ash-based geopolymer sheets manufactured by the extrusion technique. The addition of PVA fiber changed the impact failure mode from the brittle pattern to ductile pattern, resulting in a great increase in impact toughness for geopolymer composites. Reed et al. [21] showed that the addition of polypropylene fibers improved the compressive strength and ductility of geopolymer composites.

In addition to using reinforcing fibers, some researchers have also used polymer powders to modify the geopolymer. Colangelo et al. [22] studied the properties of geopolymer composites made by adding epoxy resin. The research results of Kamalloo et al. [23] showed that the addition of phenolic resin can improve the compressive and flexural strength of geopolymer by 30% and 65%, and the optimal amount of phenolic resin was 12 wt.%. Zhang et al. [24] investigated the effects of five kinds of water-soluble organic polymers on the mechanical and physical properties of uncalcined kaolinite geopolymer. It was found that the addition of polyacrylic acid and sodium polyacrylate can significantly improve the compressive strength (maximum: 29.0%) and flexural strength (maximum: 64.9%).

Among the above reinforcing materials, PVA, as a water-soluble resin, has good interfacial compatibility with geopolymer, its cost is low, and it is especially suitable for addition to geopolymer systems as a toughening material. Although some researchers have studied PVA fiber-reinforced geopolymers, the toughening mechanism has not been described in detail. Different forms of PVA resins have different strengthening and toughening mechanisms for geopolymer, which need to be further studied.

In this work, two forms of PVA powder and reinforcing fiber are used. Granular blast furnace slag with different mass ratios was firstly added into the fly ash geopolymer; the appropriate ratio was selected and used as the experiment object. In order to overcome the shortcomings of poor toughness of unmodified geopolymer, two different forms of PVA (powder and fiber) were added to the geopolymer matrix and then cured at 7 days and 28 days. The effects of PVA content, PVA morphology, and curing time on the mechanical properties of geopolymer composites were investigated. The mechanism underlying the improved mechanical properties of PVA powder and PVA fiber was analyzed, and the freeze–thaw resistance of the sample was also analyzed. This work can provide an efficient and feasible method for improving the toughness of geopolymer.

## 2. Materials and Methods

### 2.1. Materials and Properties

**Figure 1:**
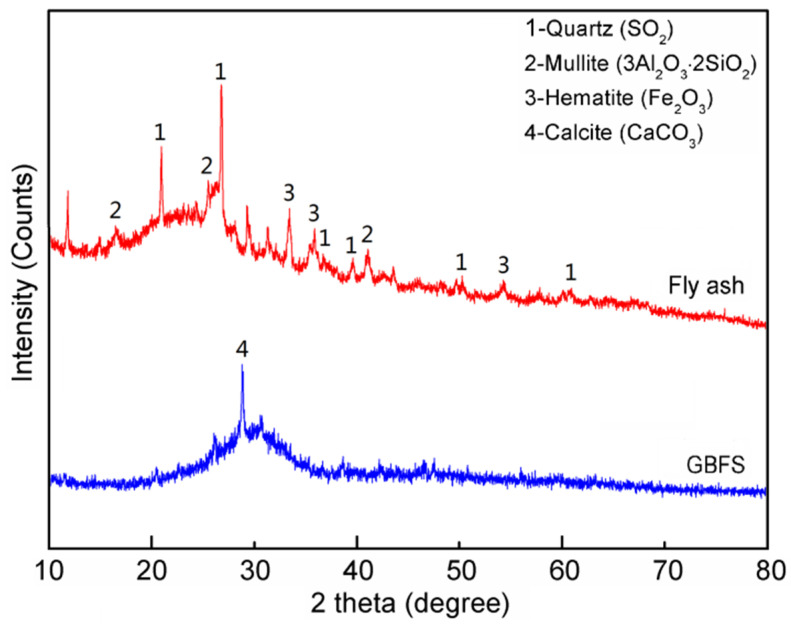
XRD analysis of the fly ash and GBFS.

The fly ash used in this study was kindly supplied by Shandong Huazhiye New Materials Technology Co., Ltd. in China. Table 1 presents the chemical composition of the fly ash determined by an X-ray fluorescence (XRF) spectrometer (XRF-1800, ShiMadzu, Kyoto, Japan). The CaO content of fly ash was only 6.00%; therefore, the fly ash was classified as a low calcium class F. The main crystalline components of fly ash were quartz, mullite, and hematite, analyzed by X-Ray Diffraction (XRD), as shown in Figure 1.

The granulated blast furnace slag (GBFS) was obtained from Henan Xiaohaibao Superfine Bead Technology Development Company, China. The main chemical components of GBFS were 38.04% CaO, 33.33% SiO_2_, and 13.76% Al_2_O_3_, as shown in Table 1. The XRD pattern in Figure 1 shows that the main crystal structures of fly ash were quartz, mullite, and hematite, and the main crystal structure of GBFS was calcite with high glass content.

Sodium hydroxide (NaOH, ≥99% by mass) was purchased from Beijing Modern Oriental Technology Development Co., Ltd. in Beijing, China. Chemical-grade sodium silicate (Na_2_SiO_3_) solution with SiO_2_/Na_2_O mole ratio of 3.2 and 37.83% solid content was provided by Beijing Hongxing Guangsha Chemical Company (Beijing, China). The alkali activator in this paper was a mixture of NaOH and sodium silicate solution. NaOH was used to adjust the SiO_2_/Na_2_O mole ratio of the alkali activator to prepare geopolymer composite samples.

Short polyvinyl alcohol fiber (PVAF) used in the work was supplied by Shandong Luke composite material Co., Ltd. (Taian, China). Specific parameters of the fiber are presented in Table 2. PVA powder (PVAP) was obtained from Sinopec Shanghai Petrochemical Co., Ltd. (Shanghai, China). Specific parameters of PVA powder are also presented in Table 3.

### 2.2. Preparation of Geopolymer Composite Samples

Alkali activator was firstly produced before the preparation of geopolymer composite samples. NaOH and Na_2_SiO_3_ solution were mixed into the alkaline activator with a SiO_2_/Na_2_O mole ratio of 1.2 and cooled to room temperature before usage. Various trials were conducted, and it was found that a SiO_2_/Na_2_O mole ratio of 1.2 provided the alkali activator with high reactivity and workability. The geopolymer slurry was easily thickened with the addition of PVA powder or fibers; thus, a water–binder ratio of 0.4 was chosen. Specific compositions of the raw materials used for the experiments are shown in Table 4.

**Figure 2:**
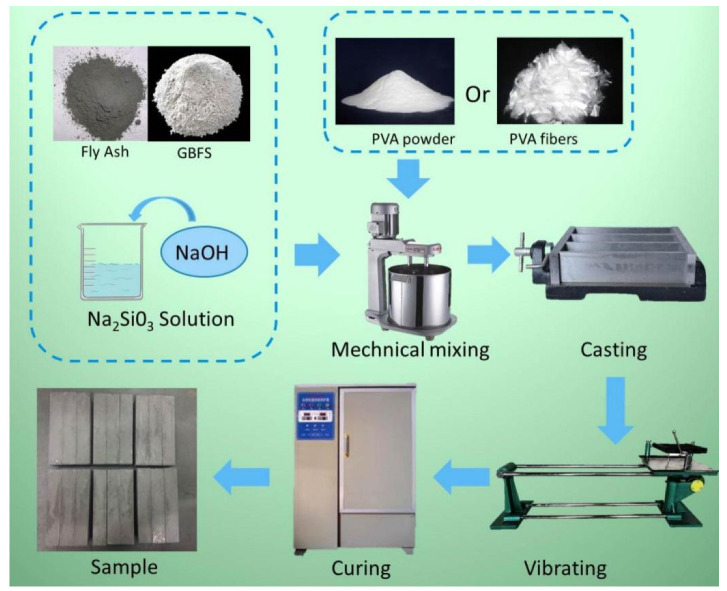
Schematic diagram of the geopolymer composite sample preparation.

Figure 2 shows the schematic diagram of the geopolymer composite sample preparation. The mixing of raw materials was carried out using a Hobart Mixer (JJ-5-type mixer, Wuxi Jianyi Instrument Machinery Co., Ltd., Wuxi, China). Firstly, GBFS and fly ash were mixed for approximately 2 min with low speed, and then water and alkaline activator solution were slowly added into the mixture and stirred at high speed for 2 min. For the specified experimental series, the PVA fibers or PVA powders were added into the wet mixture simultaneously until they were well dispersed. Subsequently, the mixture was cast into a triplet steel mold with a specimen size of 160 × 40 × 40 mm and vibrated on a shake table (ZS-15 vibrating table, Wuxi Jianyi Instrument Machinery Co., Ltd., Wuxi, China) to eliminate air bubbles. The samples were sealed with plastic films to prevent water loss and cured at room temperature for 24 h. Then, the samples were sealed with plastic bags and cured in an electric thermostatic drying oven at 65 °C for 12 h. Afterward, the samples were cured at room temperature and 95% relative humidity by a curing chamber until the mechanical test. The samples were taken out for mechanical test after 7 days and 28 days of curing.

### 2.3. Characterization

The mineralogical compositions of geopolymer composite samples were analyzed by the X’Pert Pro X-ray diffractometer (XRD, Ultima IV, Rigaku Corporation, Tokyo, Japan), using Cu Kα radiation at 40 kV power and 40 mA current. The curve was recorded in the range of 2θ = 10–80° at a scanning rate of 10.0 °/min.

Thermogravimetric analysis (TGA) tests of the samples were carried out under the air flow of 50 mL/min on TGA instrument (TGA/DSC3, Mettler Toledo Group, Greifensee, Switzerland). The temperature range was from room temperature to 800 °C and the heating rate was 20 °C/min.

Fourier-transform infrared (FTIR) spectra measurements were performed by the infrared spectrometer (Tensor 27, Brooke Fourier Infrared Spectrometer Co., Ltd., Karlsruhe, Germany). The samples were pretreated by the KBr method before testing. The resolution was 4 cm^−1^ and 32 scans were performed.

The morphology of the samples was observed by scanning electron microscopy (SEM, S-4700, Hitachi Manufacturing Co., Ltd., Hitachi, Japan). Samples were taken from the failed samples during the mechanical strength test from the parts adjoining the failure surfaces, and absolute alcohol was used to terminate the hydration. The test samples were then sufficiently dried to constant weight in a drying cabinet, and hen the sample surface was sprayed by the ion sputtering method.

The toughness of geopolymer composite samples was characterized by the compressive–flexural ratio (CFR), which is the ratio of compressive strength to flexural strength. The compressive strength and flexural strength of samples were measured with a compression testing machinery (TYE-200B, Wuxi Jianyi Instrument Machinery Co., Ltd., Wuxi, China). These tests were carried out according to GB/T 50081-2019 standard in China. Three samples were used for each experiment, and average values were calculated.

The freeze–thaw resistance properties of the samples were in accordance with the standard GB/T50082-2009, and the freeze–thaw test machine (JCD-40J, Beijing Shuzhiyilong Instrument Co., Ltd., Beijing, China) was used to perform a slow freeze test on the test samples. The freeze–thaw process underwent 25 cycles, and the mass loss and the compressive strength loss were tested.

## 3. Results and Discussion

### 3.1. Toughness Behavior

**Figure 3:**
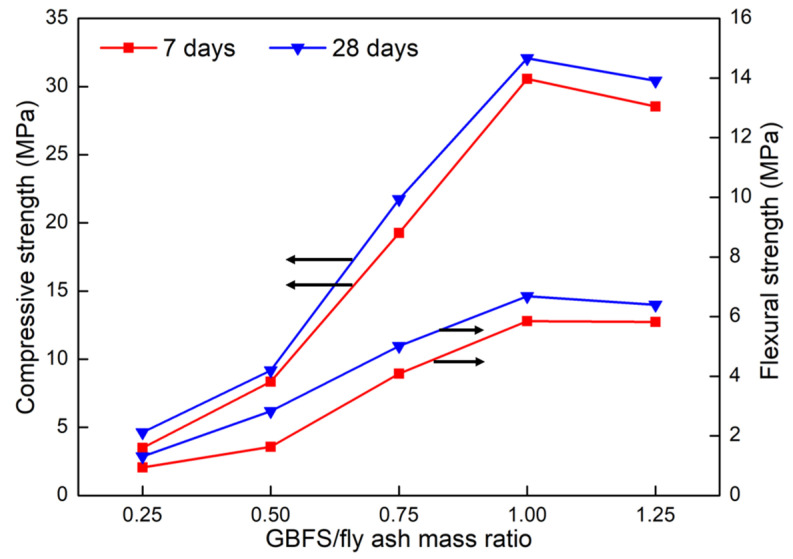
Flexural and compressive strengths of samples with different GBFS/fly ash mass ratios.

Since fly ash is a low-calcium material, the pure fly ash-based geopolymer prepared by the alkali activation method at room temperature has a long setting time and poor mechanical properties. Adding more active raw materials (e.g., GBFS) can accelerate the reaction of geopolymer, which is a feasible way to improve the mechanical properties [25,26,27]. In this section, the mechanical behavior of geopolymer composites prepared with different GBFS/fly ash mass ratios was studied. The results are shown in Figure 3.

It can be noted that the mechanical behavior of the samples cured for 28 days was better than that of the samples cured for 7 days. As the mass ratio of GBFS/fly ash increased, the compressive strength and flexural strength of the samples also showed a similar increase–peak–decrease trend. When the mass ratio of GBFS/fly ash was 1:1, the flexural strength and compressive strength of the samples both reached their peaks. After curing for 7 days and 28 days, the flexural strength of samples reached 5.85 MPa and 6.68 MPa, respectively. For the toughness of geopolymer, the flexural strength was an important evaluation criterion for the mechanical properties of geopolymer, and it was meaningful to discuss the compressive–flexural ratio under the condition of the same flexural strength [28,29]. Therefore, GBFS/fly ash mass ratio of 1:1 was selected as the object of further research.

**Figure 4:**
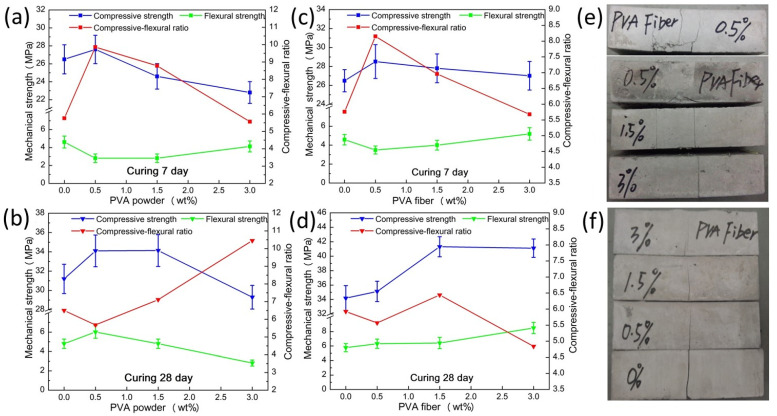
Mechanical behavior of samples with different PVA powder/fiber contents: (**a**,**b**) samples with PVA powder; (**c**,**d**) samples with PVA fiber; (**e**) samples with different PVA fibers after compressive test; (**f**) samples with different PVA fibers after flexural test.

In order to overcome the shortcomings of insufficient toughness of unmodified geopolymers and to explore the toughening mechanism of PVA with different morphologies, PVA powder and PVA fibers were added to geopolymer composites for investigation. Figure 4 shows the mechanical properties of geopolymer composites with different PVA powder and fiber contents.

The ratio of compressive strength to flexural strength (C/F; Figure 4) is usually used by researchers to characterize the toughness of cement materials [30]. As can be seen from Figure 4a, when the curing time was 7 days, the addition of PVA powder did not significantly improve the compressive strength and flexural strength of the geopolymer, and it even decreased when the content of PVA powder was 1.5 wt.%. After the curing time was extended to 28 days, when the content of PVA powder was 0.5 wt.%, the compressive strength and flexural strength increased from 31.21 MPa and 4.8 MPa to 34.12 MPa and 6.01 MPa, respectively, as shown in Figure 4b. However, this was not as significant as the improvement in mechanical behavior brought by the addition of PVA fiber. The mechanical strength of samples with different PVA fibers content was also improved with the extension of the curing time, especially the compressive strength, as shown in Figure 4c,d. Under the condition of 28 days of curing, the compressive strength reached the maximum at a fiber content of 1.5 wt.%, which was 41.31 MPa, and the flexural strength at this time was 6.41 MPa. The maximum flexural strength was 8.49 MPa when the fiber content was 3 wt.%, and the compressive strength was 41.11 MPa. Although the compressive strength of the sample with 3 wt.% fiber content was slightly lower than that of the sample with 1.5 wt.% fiber content, the flexural strength of the former was 32.44% higher than that of the latter, and the C/F ratio was the minimum. It can be considered that the best mechanical strength was achieved with a fiber content of 3 wt.% after 28 days of curing. Therefore, when the PVA fibers and PVA powders in the sample were optimized are cured for 28 days, the toughening effect of the PVA fiber was better than that of the PVA powder.

Figure 4e,f show specimens taken from the failed samples with different PVA fiber contents after the compressive strength and flexural strength tests. The sample with 0.5 wt.% PVA fiber content had many cracks on the surface. However, the samples with PVA fiber content of 1.5 wt.% and 3 wt.% did not exhibit significant changes in the pressure surface; moreover, they maintained the original morphology and had residual strength. Figure 4f shows that the sample without PVA fiber was completely broken, and the cracks became shorter and narrower with the increase in fiber content.

### 3.2. Freeze–Thaw Resistance Properties

**Figure 5:**
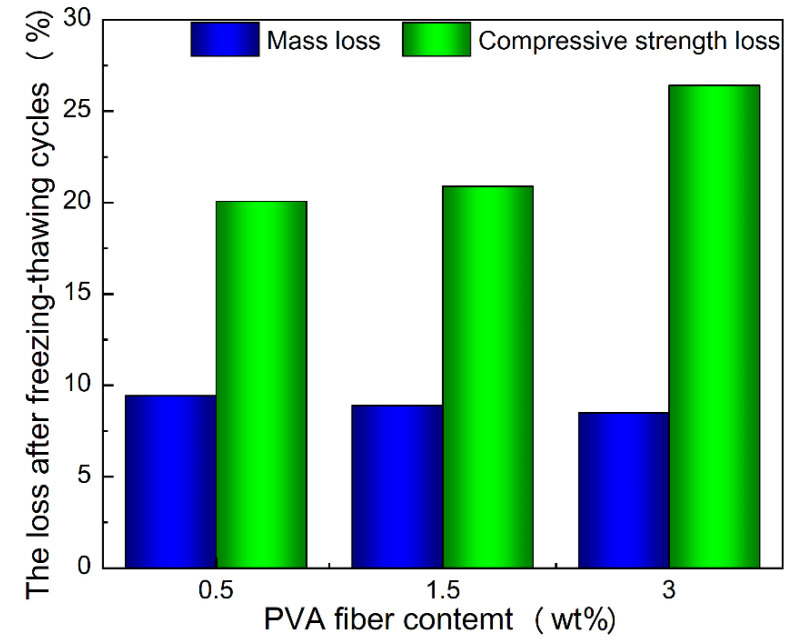
The loss of geopolymer composite samples modified with PVA fiber after freezing–thawing cycles.

The freeze–thaw cycle resistance is a key indicator for the performance of geopolymer. Figure 5 shows the mass and strength loss of geopolymer composite samples with different PVA fiber contents after 25 freeze–thaw cycles. After freeze–thaw cycles, the mass loss of all samples was less than 10%. Among them, the sample with 3 wt.% PVA fiber had the least mass loss. The compressive strength loss decreased with the increase in PVA fiber content and was the lowest with 3 wt.% PVA fiber content, which was 20.65%. According to the GB/T 50082-2009 standard, a sample with compressive strength loss less than 25% is satisfactory. The freeze–thaw resistance of the sample with 3 wt.% PVA fiber was far lower than this index. Therefore, according to the freeze–thaw test results, the geopolymer toughened by PVA fiber can meet the freeze–thaw requirements.

### 3.3. XRD Analysis

**Figure 6:**
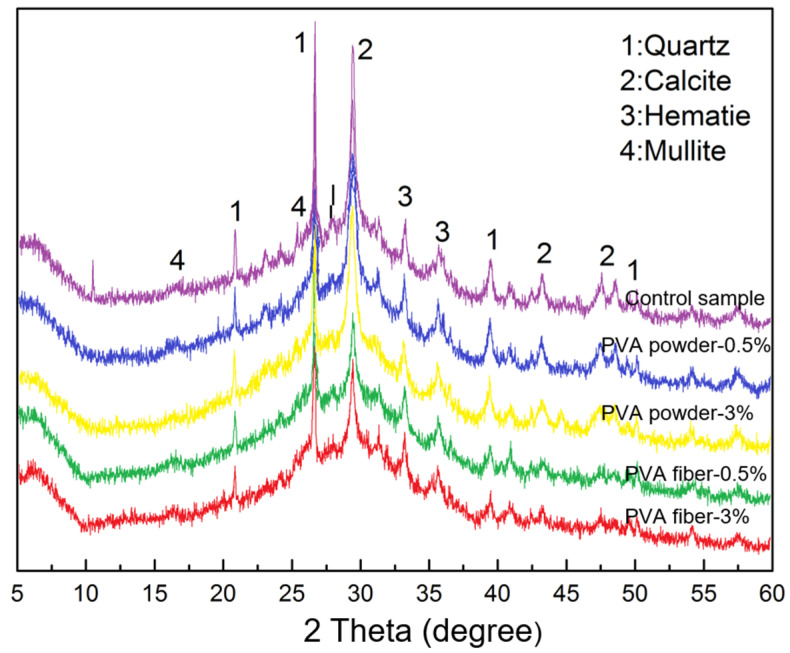
XRD patterns of PVA modified geopolymer composites.

In order to demonstrate whether the addition of PVA would affect the alkali-induced reaction of geopolymer, XRD analysis was carried out. Figure 6 shows the XRD pattern of the geopolymer samples modified with PVA fibers and powders. It indicated that the control sample and the PVA-toughened samples had high diffusion peaks at 20–35°, and the peak shapes of the samples containing different PVA content were consistent. The addition of PVA would not change the mineral composition of GBFS/fly ash geopolymer and had nothing to do with the form of PVA. Quartz, hematite, mullite, calcite, and fly ash of GBFS did not participate in the reaction and constituted the main crystal structure [31,32]. This indicated that the addition of PVA had no significant impact on the reaction process of geopolymer, and no new phases were produced, which mainly existed as reinforcing phases in geopolymer.

### 3.4. IR Analysis

**Figure 7:**
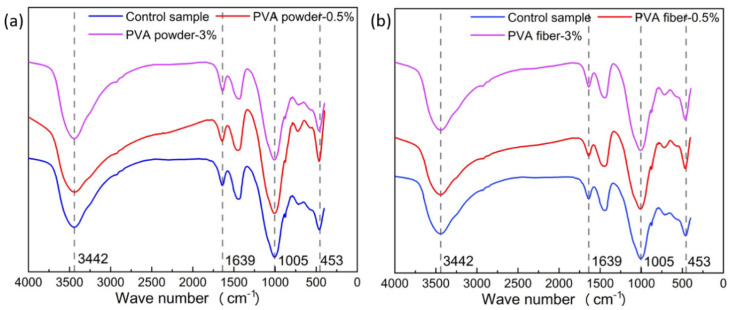
IR analysis of PVA-modified geopolymer composite samples: (**a**) PVA powder; (**b**) PVA fiber.

Through infrared spectroscopy analysis, the types of functional groups and the role of PVA in geopolymers were further investigated. The infrared spectrum of each sample is shown in Figure 7. The results showed that the absorption bands corresponding to the wavenumbers of 3442 cm^−1^ and 1639 cm^−1^ were caused by the bending vibration and stretching vibration of –OH [33]. The absorption bands of 453 cm^−1^ and 1005 cm^−1^ corresponded to the Si–O–Si stretching vibration of the SiO_4_ tetrahedron and the O–Si–O bending vibration, respectively [34]. There was no significant difference in the IR spectra of the samples, while a slight wavenumber difference in the position of the absorption band illustrated that the addition of PVA did not change the functional groups in the geopolymer. It can be considered that PVA did not alter the functional types of geopolymer, and the components only relied on the combination of intermolecular van der Waals forces.

### 3.5. TGA Analysis

**Figure 8:**
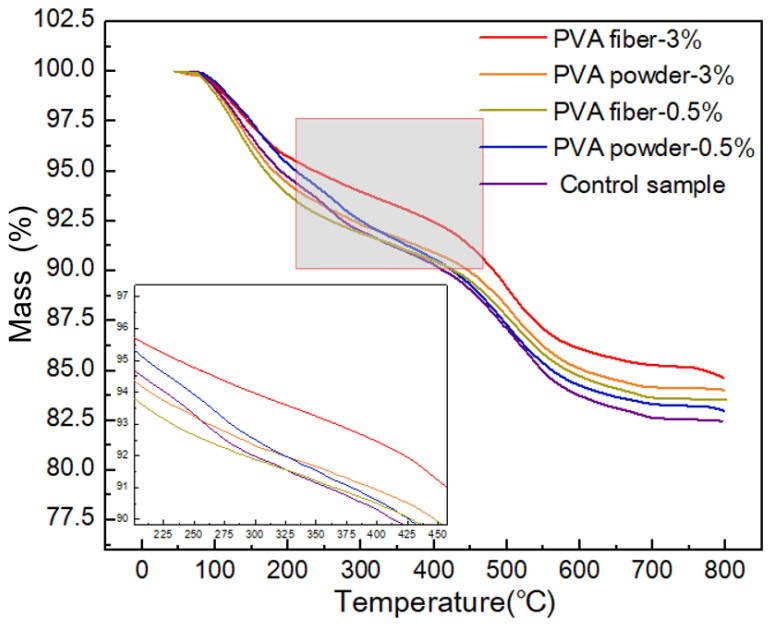
TGA analysis of PVA modified geopolymer composites.

To explore the influence of the amount and form of PVA on the thermal stability of geopolymer, thermogravimetric analysis was performed on different samples. Figure 8 shows the TGA patterns of specific geopolymer composite samples with PVA.

The first quality reduction process was related to dehydration. All samples had 7–9% quality loss in the range of 0–150 °C. This process was related to the evaporation of free water present on the surface of the sample or in the pores. Then, all samples lost weight completely at about 650 °C, including the loss of bound water and the decomposition of polyvinyl alcohol, accounting for about 8% of the total mass. As the control sample had no polyvinyl alcohol added, as the temperature continued to rise, the final mass was reduced by 17.5%. The final masses of samples with 0.5 wt.% and 3 wt.% PVA water-soluble powders were reduced by 16% and 16.8%, respectively. The final masses of samples with PVA fiber content of 0.5 wt.% and 3 wt.% were reduced by 15.5% and 16.5%, respectively. By comparison, it can be found that the addition of polyvinyl alcohol could reduce the thermal weight loss of GBFS/fly ash based geopolymer, and, as the PVA content increased, the thermal stability of the modified geopolymer was enhanced. It can also be seen that the effect of PVA fiber in developing thermal stability in geopolymer was better than that of PVA powder. This may be because the surface of polyvinyl alcohol contained hydrophilic active hydroxyl groups, which could form hydrogen bonds with water molecules and delay their evaporation.

### 3.6. SEM Analysis

**Figure 9:**
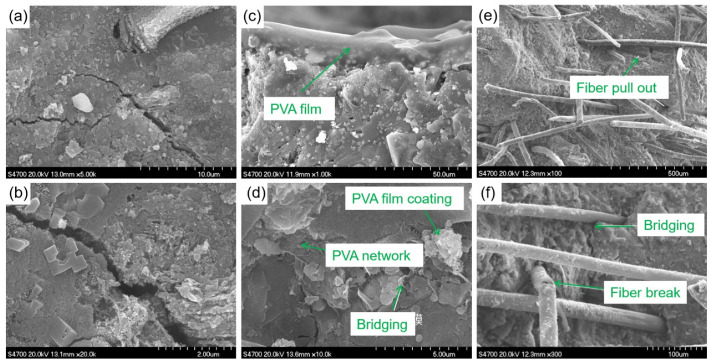
SEM micrographs of samples: (**a**,**b**) control sample; (**c**,**d**) PVA powder-modified samples; (**e**,**f**) PVA fiber-modified samples.

The toughening mechanism of PVA fibers and PVA powders and its effect on the mechanical strength were studied. The microstructure of the geopolymer composite samples was observed; Figure 9 presents the SEM micrographs of samples.

It can be seen from Figure 9a,b that there were interlaced small cracks inside the control sample. These cracks would continue to expand when damaged by external forces, weakening the mechanical strength of the sample. Figure 9c shows the microstructure of the sample with 0.5 wt.% PVA powders. The results illustrated that the PVA powder was dissolved in water during the preparation process, dried, and formed into films in the subsequent curing process. Although some researchers [35] believe that these films can bond the interface, reduce the interface stress, and increase the interface energy, from our test results, PVA powder provided almost no improvement in mechanical properties. In addition, upon increasing the content of PVA powders to 3 wt.%, the microstructure of the geopolymer composite sample was as shown in Figure 9d. However, too much PVA powder led the PVA film to wrap the raw materials, which hindered the completion of further reactions, resulting in internal defects. This can also explain the macro-mechanical performance degradation caused by the high content of PVA powders. The micro-morphology of 3 wt.% PVA fiber-modified geopolymer composite sample can be seen from Figure 9e,f. PVA fibers played a variety of different roles in the sample during the destruction process. There was a strong interface bond between the fiber and the geopolymer, which consumed a lot of destruction energy during the separation process. During the destruction process, cracks appeared in the material, and some fibers could still be well combined with the matrix. From Figure 9f, it can also be observed that cracks began to appear inside the material, and, after encountering the fiber, they grew along the interface between the fiber and the matrix, and crack deflection occurred. Fibers can also bridge the cracks and limit their growth, which is consistent with the macroscopic phenomena shown in Figure 4e,f. Due to fiber pull-out, breakage, bridging, and crack deflection, the material needs to consume a lot of energy during the destruction process [36]. The failure mode of the PVA fiber-toughened geopolymer composite also changed from brittle fracture to toughness fracture, as also reflected in the results of the mechanical strength test.

## 4. Conclusions

In this work, different contents of PVA powder and PVA fiber were added to toughen geopolymer composites, and the effects of PVA morphology and content on the properties of GBFS/fly ash geopolymer were studied to reveal the toughening mechanism of different forms of PVA. The research results of this paper can be applied in the field of building materials. The following conclusions can be drawn:

- The addition of GBFS can effectively improve the mechanical strength of fly ash geopolymer, and the mechanical strength was best when the ratio of GBFS to fly ash was 1:1 after curing for 28 days.
- PVA did not change the composition of geopolymer, nor did it react with the reaction process. The addition of PVA powders did not significantly improve the mechanical strength of the geopolymer. However, the addition of PVA fibers could effectively improve the mechanical strength. The optimal fiber content was 3 wt.%, resulting in compressive strength and flexural strength values of 41.11 MPa and 8.49 MPa, respectively, after curing for 28 days.
- The main reason that PVA powder did not significantly improve the mechanical properties of the matrix was that the powder formed films after being dissolved in water, which hindered the alkali-induced reaction. The addition of PVA fiber would not damage the continuity of the matrix and would form a bridge inside the matrix to compensate for internal defects. When the geopolymer was externally damaged, it consumed a large amount of energy through fiber pulling, breaking, etc., and it had an inhibitory effect on crack propagation.

## Figures and Tables

**Table 1:** The main chemical composition of fly ash and GBFS.

Chemical Composition (%)	Fly Ash	GBFS
SiO_2_	45.43	33.33
Al_2_O_3_	35.89	13.76
Fe_2_O_3_	4.32	0.62
CaO	6.00	38.04
SO_3_	4.11	2.90
TiO_2_	1.63	0.78
MgO	0.66	9.23
K_2_O	0.55	0.34
P_2_O_5_	0.51	0.02
Na_2_O	0.14	0.32

**Table 2:** Specific parameters of PVA fiber.

Parameter	Diameter (μm)	Length (mm)	Tensile Strength (MPa)	Young’s Modulus (GPa)	Elongation (%)	Density (g/cm^3^)
Value	20	6	1200	35	6–11	1.30

**Table 3:** Specific parameters of PVA powder.

Parameter	Mesh Number	PH	Viscosity (mPa·s)	Molecular Weight	Degree of Polymerization
Value	120	5–7	20.0–26.5	72,600–81,400	1650–1850

**Table 4:** Experimental arrangement for raw material ratio investigation.

Experiment Number	SiO_2_/Na_2_O Mole Ratio	Activators/Ash Mass Ratio	GBFS/Ash Mass Ratio	PVA Powder (wt.%)	PVA Fiber (wt.%)	Water/Binder Mass Ratio
1	1.2	0.46	0.25	-	-	0.4
2	1.2	0.46	0.5	-	-	0.4
3	1.2	0.46	0.75	-	-	0.4
4	1.2	0.46	1.0	-	-	0.4
5	1.2	0.46	1.25	-	-	0.4
6	1.2	0.46	1.0	0.5	-	0.4
7	1.2	0.46	1.0	1.5	-	0.4
8	1.2	0.46	1.0	3	-	0.4
9	1.2	0.46	1.0	-	0.5	0.4
10	1.2	0.46	1.0	-	1.5	0.4
11	1.2	0.46	1.0	-	3	0.4
